# Application of Polovodova’s method for the determination of physiological age and relationship between the level of parity and infectivity of *Plasmodium falciparum* in *Anopheles gambiae s.s*, south-eastern Benin

**DOI:** 10.1186/s13071-015-0731-7

**Published:** 2015-02-22

**Authors:** Rodrigue Anagonou, Fiacre Agossa, Roseric Azondékon, Marc Agbogan, Fréderic Oké-Agbo, Virgile Gnanguenon, Kèfilath Badirou, Ramziath Agbanrin-Youssouf, Roseline Attolou, Gil Germain Padonou, Arthur Sovi, Razaki Ossè, Martin Akogbéto

**Affiliations:** Centre de Recherche Entomologique de Cotonou, 06 BP 2604 Cotonou, Benin; Faculté des Sciences et Techniques, Université d’Abomey-Calavi, Calavi, Bénin; University of Wisconsin, Milwaukee, WI USA

**Keywords:** *Anopheles gambiae s.s*, Parity level, Classical dilaceration, Oil injection, Infectivity

## Abstract

**Background:**

Polovodova method based on counting follicular dilatations estimates the number of egg-laying in mosquitoes. However, some researchers doubt the reliability of this method because of the absence of multiple dilatations in vectors after many gonotrophic cycles. It is in this context of controversy that our study was carried out to evaluate the importance of follicular dilatations in the determination of parity levels in *An. gambiae s.s.* Moreover, the application of this method allowed us to clarify the evolution of vectors’ infectivity to *P. falciparum* according to their parity level.

**Methods:**

We used two techniques to determine the parity level in *An. gambiae s.s*. We used two batches of wild strain mosquitoes reproduced after a known number of egg-laying in laboratory. The first batch was submitted to oil injection in the ovaries using a micropipette. In the same way, the classic technique of ovaries dilaceration (a technique based on the Polovodova method) was applied to the second batch. In order to assess relationship between parity level and mosquitoes’ infectivity, Polovodova method was applied on vectors collected on humans. Finally, Heads and thoraces of these vectors were individually analyzed for *P. falciparum* antigen detection using an ELISA assay.

**Results:**

In the first batch including 50 female mosquitoes “never laid”, 50 “laid once”, 50 “laid twice” and 48 “three times”, oil injection technique revealed 42 nulliparous, 44 uniparous, 46 biparous and 44 triparous respectively. Overall, Polovodova method was effective using oil injection technique (p > 0.05). On the other hand, in the second batch that has a similar number of laying to the first batch, the application of Polovodova method through classical technique of ovaries dilaceration was ineffective with multiparous females (p < 0.05). Moreover, probability of vector infectivity increased with the number of egg-laying (p < 0.0001).

**Conclusion:**

Our results revealed that the Polovodova method is reliable for estimating the number of egg-laying in *Anopheles gambiae s.s.* using oil injection technique in the ovaries. The study has also showed an increased likelihood of infectivity in vectors according to the number of egg-laying.

**Electronic supplementary material:**

The online version of this article (doi:10.1186/s13071-015-0731-7) contains supplementary material, which is available to authorized users.

## Background

The physiological age of insects, vectors of disease, was assessed by several methods [1]. These methods included ovarian tracheoles [2], decreased fecundity [3], copulation index [4], copulation marks [5], fat modifications [6], cuticular changes [7], appearance of Malpighian tubules [8,9], intestinal changes [10], accumulation of fluorescent compounds in specific cells [11], wing tear, and scales removal in mosquitoes [12,13]. Likewise, it is assumed that after each egg-laying, dilatation is observed at the region of follicular tube where the egg originated from. In theory, the number of dilatations in the ovarioles (follicles) must equal to the number of egg-laying in female mosquitoes – which is an indicator of physiological age [14,15]. In tropical Africa, this method has been widely used to determine the physiological age of malaria vectors [16].

Several studies reported the existence of several dilatations in multiparous *An. gambiae* and concluded that the method of Polovodova [14] is applicable to this species in order to estimate their number of egg-laying [17-19]. However, the absence of multiple dilatations was also observed in the same species after several egg-laying [20-22]. These differences explain some reservations observed by some researchers in the use of the method of Polovodova for the determination of number of egg-laying (parity level) in mosquitoes. It is in this context of controversy among different authors that this study was initiated. Furthermore, the application of results of Polovodova method for physiological age determination allowed us to verify if the likelihood of infectivity in *An. gambiae* to *Plasmodium falciparum* is higher in older females. In order to count the number of follicular dilatations per egg-laying, Polovodova method was applied to females of *An. gambiae s.s.* reared in laboratory after larval collection. The relationship between physiological age and infectivity of vectors to *P. falciparum* was verified in female adult of *An. gambiae s.s.* collected by human landing catch.

## Methods

### Study area

**Figure 1:**
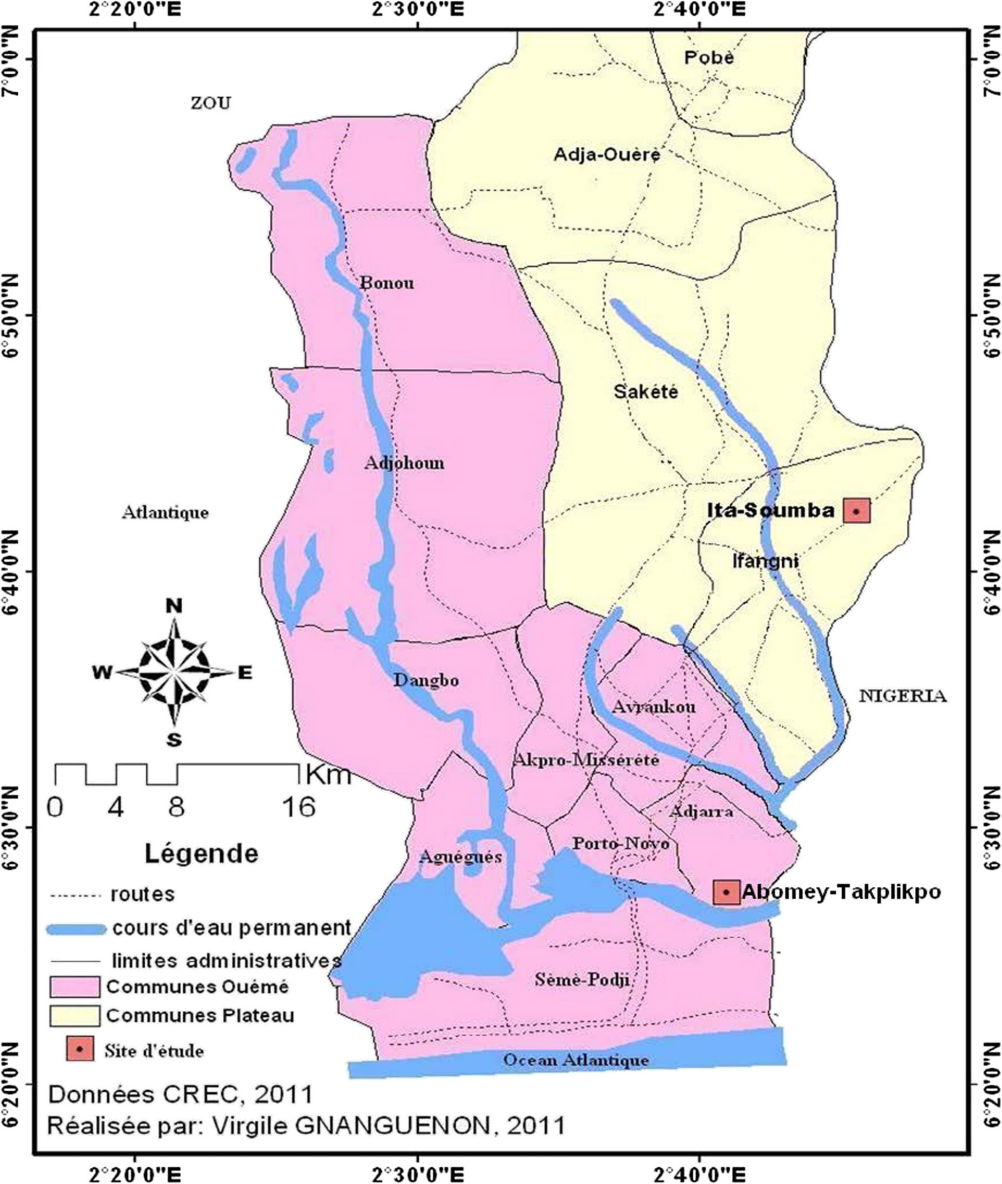
**The villages of Itassoumba (district of Ifangni) and Abomey-takplikpo (district of Adjarra) in South-Eastern Benin.**

This study was carried out at Adjarra and Ifangni districts in Benin (Figure 1).

### Adjarra

In Adjarra district, located 06°27’00’ N and 01°56’00’ E, the study was conducted in the village of Abomey-takplikpo (Figure 1). Adjarra is located in the department of Oueme with a population of 60,112 inhabitants and an area of 112 km^2^ [23]. It is bordered in the north by Avrankou district, in the south by Seme-Podji district, in the west by Porto-Novo district and in the east by Nigeria. The climate is sub-equatorial with two dry seasons and two rainy seasons. The average rainfall is 1200 mm per year. The hydrographic network of this district includes the lagoon of Porto-Novo in the south and Aguidi River in the northeast. The vegetation is sparse; it is composed of shrubs, grass and by sacred forests relics.

### Ifangni

Entomological surveys were conducted at Itassoumba (Figure 1) in the district of Ifangni (Province of Plateau) located 06°38’56’N and 02°43’14’E, with a population of 71,606 inhabitants and an area of 242 km^2^ [23]. The climate is Guinean with two dry seasons and two rainy seasons. The annual precipitation is between 800 mm and 1400 mm. The vegetation includes sacred forest relics, plantations of oil palms, shrubs and tall grass. Itassoumba is crossed by swamps. In the dry season, the breeding sites of *An. gambiae s.l.* are scarce. However, breeding sites are particularly permanent in Itassoumba due to the presence of fish ponds and vegetable farming. Once animal feed reserved for small fry and fish are present on the surface of the water, they constitute a source of food favoring the proliferation of malaria vectors.

### Study on wild *Anopheles gambiae s.s.* reared at insectarium

#### Sampling of *An. gambiae s.s*. larvae

Collection of larvae was only conducted in Itassoumba because of the permanent presence of *An. gambiae s.s.*breeding sites [24,25]. This sampling was performed according to the method of “Dipping” with ladles fitted with long handles, plastic buckets, cups, bottles, containers and a filter cloth.

#### Rearing of larvae

At the Insectarium, larvae were placed in trays containing breeding site water. They were distributed on average in lots of 100 per tray to not only optimize their growth, but also to avoid cannibalism. The use of breeding site water, was to avoid the influence of chemical residues such as sodium hypochlorite (NaOCl) on larval growth. The larvae were fed with croquette of cat (5 g mixed in 500 ml of breeding site water for at least 80 larvae). Each tray was covered with an untreated net and stored in insectarium conditions (relative humidity ranges from 70-80% with a temperature between 25 and 30 degrees). Photoperiodicity was assured by fluorescent lights. After emergence, adult mosquitoes were collected and put in a cubic cage. Females were isolated in a cage and fed with 10% honey solution.

### Induction of egg-laying in *Anopheles gambiae*

Females aged from 5 to 6 days were fed with blood from guinea pigs, placed in cages at 19 hours and removed the next morning at 7 hours. Fed females were placed in other cages and were re-fed two days later, after complete digestion of the first blood meal. The additional blood meal was use to complete the maturation of ovaries in nulliparous females. Females that have taken two blood meals were put on an individual egg-laying. A sample of blood fed nulliparous and parous mosquitoes was dissected at different stages of ovarian development [26].

For individual egg-laying, female of *An. gambiae s.s.* was put in a cup covered by a piece of untreated net. A nest box was placed at the bottom of each cup (cotton swab moistened with water upon a Whatman paper of 5 cm radius). Mosquito was fed with 10% honey solution every day. This served as food for gravid females in experimentation. After the first egg-laying, a sample of uniparous mosquitoes was dissected after 24 hours of observation. The observation time allowed mosquitoes to release any residual eggs.

The remaining uniparous mosquitoes, once released into cage, were blood fed again for the second egg-laying round. After the observation period, a sample was also dissected. The experiment was repeated on the remaining biparous mosquitoes to obtain triparous mosquitoes.

### Dissection of the ovaries and determination of the physiological age of reared mosquitoes

Polovodova method was applied with oil injection technique on a batch of 198 mosquitoes divided into four samples of known ages. The ovaries of each mosquito were dissected on a slide in a physiological liquid (0.9% Natrichlorid + Neutral Red 1/5000 to 1/3000) using a binocular microscope. The ovaries were extracted carefully while maintaining the common and lateral oviducts. Paraffin oil was injected into the ovaries through the common oviduct using a glass micropipette with an opening of 0.05 μm at the tip. At this step, we observed swelling of the ovary which had received paraffin oil. The colored ovary was carefully opened from its dorsal face using dissecting needles. The samples were mounted onto a slide and read under an incorporated (4X-10X) camera microscopy. Thus, the maximum number of dilatations (indicator of physiological age) carried by the ovarioles was recorded.

Similarly, the same Polovodova method was applied with the classic (conventional) ovaries dilaceration on 200 mosquitoes divided in four samples of known ages. When the ovaries were dissected in the physiological liquid, ovarioles are simply and carefully isolated with dissecting needles. The maximum number of dilatations carried by the ovarioles was also recorded.

### Study on adults *Anopheles gambiae s.s.* collected by human landing catch

#### Sampling of adult mosquitoes and determination of physiological age

Mosquitoes were caught on man from 9:00 p.m. to 5:00 am. Collections were carried out inside and outside houses using hemolytic tubes and torch. After each collection, *An. gambiae s.l.* were identified morphologically [27,28]. Their physiological age was determined by the Polovodova method [14] applied with oil injection technique.

### Conservation of biological materials

Dissected mosquitoes were divided in two parts (head-thorax and carcass) and stored in Eppendorf tubes containing silica gel at −20° C. Each Eppendorf tube was labeled (date, place of capture and order number of mosquito).

### Infectivity of vectors in *Plasmodium falciparum* and identification of *An. gambiae s.l.* species

Head-thorax of each vector was used to detect circumsporozoïtic antigen of *P. falciparum* (CSP) by enzyme-linked immunosorbent assay (ELISA) using monoclonal antibodies against the CSP [29]. The carcasses were used individually for species identification by Polymerase Chain Reaction (PCR) [30].

### Statistical analysis

Mid-p tests and Fisher’s Exact [31] were used to assess the effectiveness of oil injection techniques and classical dilacerations of ovaries for determination of the number of egg-laying in mosquitoes. The odds ratio was estimated using the unconditional method of maximum likelihood (Wald). Confidence intervals were estimated using the estimation of normal approximation (Wald) method. The effect of Christophers’ ovarian development stages on the effectiveness of physiological age determination techniques was assessed using logistic regression. The non-conformity indicator factor to the actual age of mosquito as endogenous factor and indicator of Christophers’ ovarian development stages was taken into account as exogenous factor, followed by an analysis of deviance with the likelihood ratio test [32]. The method of definition of “contrasts” allowed us to compare the influence of Christophers’ stages on the effectiveness of physiological age determination techniques. Poisson’s regression was used to assess the relationship between infectivity and physiological age of mosquitoes. The likelihood ratio test was used to estimate the significance level of the relationship. Statistical tests were performed using R-2.15.2 software [33].

### Ethical consideration

Ethical approval for this study was received from the Ministry of Health [N°007/2010]. Mosquito collectors gave prior informed consent and they were vaccinated against yellow fever. They were also subjected to regular medical check-ups with preventive malaria treatments.

## Results

### Structure of observed age in anopheles after oil injection and conventional ovaries dilaceration

Polovodova method was applied with conventional ovaries dilaceration on lot 1 and with oil injection on lot 2 of mosquitoes at different ovaries development stages (Additional file 1).

### Structure of ovariole in nulliparous and parous females *Anopheles gambiae s.s.*

**Figure 2:**
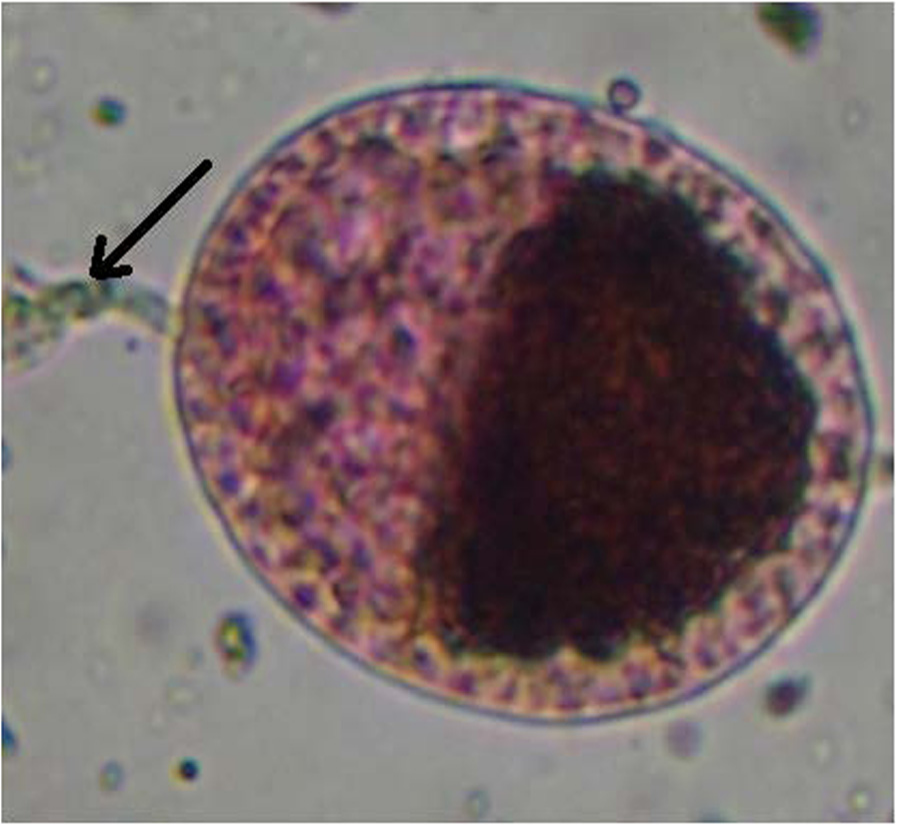
**Ovariole devoid of dilatation observed from oil injection technic (Photo CREC, 2013).**

**Figure 3:**
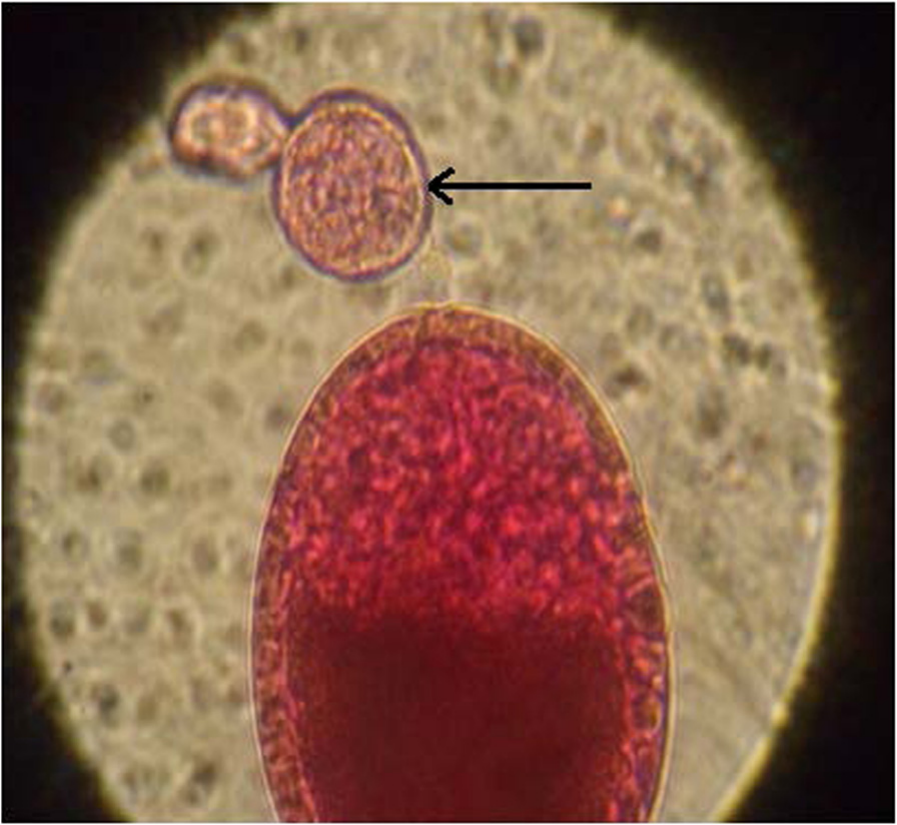
**Ovariole with one dilatation observed from oil injection technic (Photo CREC, 2013).**

**Figure 4:**
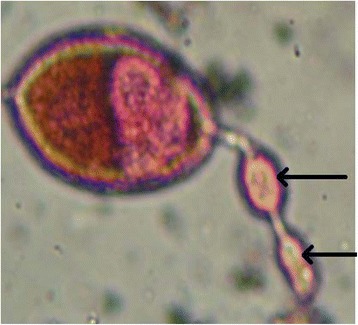
**Ovariole with two dilatations observed from oil injection technic (Photo CREC, 2013).**

**Figure 5:**
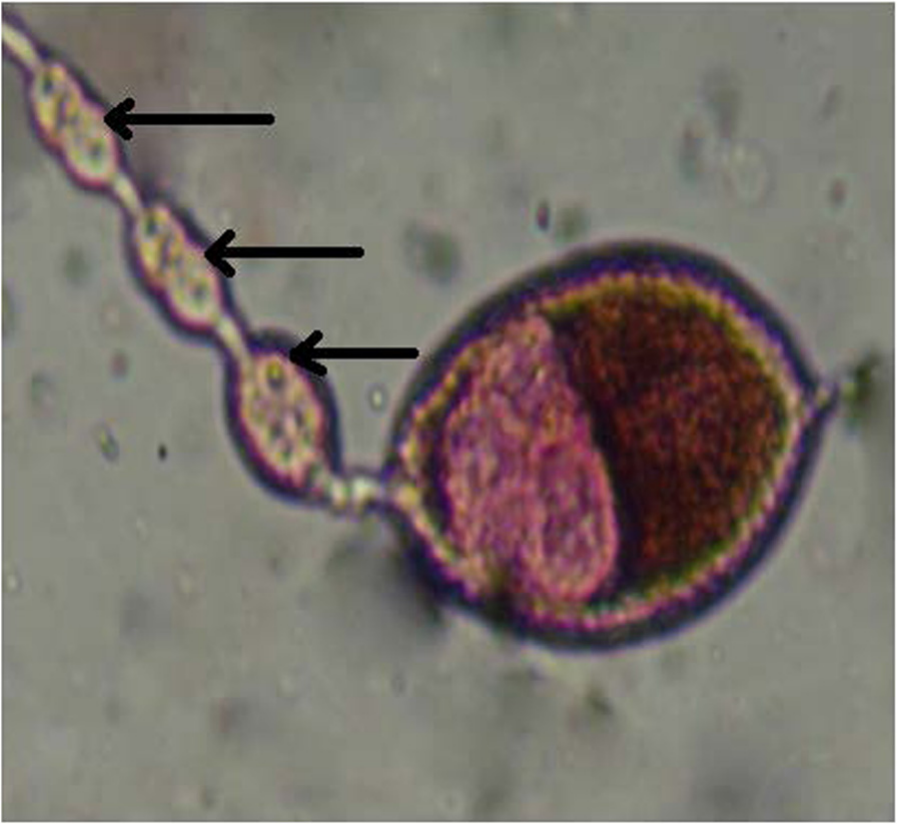
**Ovariole with three dilatations observed from oil injection technic (Photo CREC, 2013).**

Polovodova method applied with oil injection technique and conventional ovaries dilaceration revealed several types of ovarioles in nulliparous and parous *An. gambiae s.s.* females. Overall, we observed ovaries with no dilatation (Figure 2) and ovarioles with one (Figure 3) or several dilatations (Figures 4 and 5). Mosquitoes with one, two or three dilatations were those that laid eggs once (uniparous), twice (biparous) and three times (triparous). However, those with no dilatation are nulliparous.

### Efficacy of conventional ovaries dilaceration for the determination of the number of egg-laying in females *Anopheles gambiae s.s.*

In total, 200 mosquitoes where determined physiological age using Polovodova conventional ovaries dilacerations were examined (Table 1). These mosquitoes were divided into four samples of 50 mosquitoes (respectively nulliparous, uniparous, biparous and triparous). The conventional ovaries dilaceration technique was effective in the determination of nulliparous and uniparous mosquitoes. With this technique, physiological age of 38 nulliparous females and 42 uniparous was confirmed (Table 1). However, this technique showed its inefficacy with multiparous mosquitoes. On 50 biparous mosquitoes, the technique just recognized 7 (p < 0.0001). Only one mosquito was revealed triparous on the 50 dissected using the conventional ovaries dilaceration (p < 0.0001). This result suggests that conventional ovaries dilaceration is not applicable to the determination of the number of egg-laying in *Anopheles gambiae s.s.*

**Table 1:** **Efficacy of conventional ovaries dilaceration for determination of the number of egg-laying in** ***Anopheles gambiae s.s***

**Age**	**Sample**	**Nb conformity**	**Total mosquito**	**% conformity**	**OR**	**IC-95%(OR)**	**P**
p0	Control	50	200	25	1.00	-	-
	CDT	38	200	19	0.70	[0.44-1.13]	0.184
p1	Control	50	200	25	1.00	-	-
	CDT	42	200	21	0.79	[0.50-1.27]	0.406
p2	Control	50	200	25	1.00	-	-
	CDT	7	200	3,5	0.11	[0.04-0.25]	<0.0001
p3	Control	50	200	25	1.00		
	CDT	1	200	0,5	0.02	[0.002-0.01]	<0.0001

CDT: Conventional ovaries dilaceration technique, p0: nulliparous, p1: uniparous, p2: biparous, p3: triparous, Nb: number, IC: Confidence interval, p: p-value, OR: odds ratio.

### Efficacy of oil injection technique into ovaries for determination of the number of egg-laying in females *Anopheles gambiae s.s.*

One hundred and ninety eight *An. gambiae s.s.* was examined with Polovodova oil injection technique (Table 2). This technique was applied on 4 samples representing nulliparous mosquitoes (n = 50), uniparous (n = 50), biparous (n = 50) and triparous (n = 48). Overall, the physiological age was confirmed in respectively 42 nulliparous mosquitoes, 44 uniparous, 46 biparous and 44 triparous. No significant difference was observed between the number of egg-laying and the number of dilatations observed on the ovarioles regardless of the level of parity. Oil injection technique in ovaries is more likely applicable for the determination of the number of egg-laying in *An. gambiae s.s*. However, in this study, we were unsuccessful in determining the physiological age of all mosquitoes examined.

**Table 2:** **Efficacy of oil injection technique into ovaries for the determination of the number of egg-laying in** ***Anopheles gambiae s.s***

**Age**	**Sample**	**Nb conformity**	**Total mosquito**	**% conformity**	**OR**	**IC-95%(OR)**	**P**
p0	Control	50	198	25,25	1.00	-	-
	OIT	42	198	21,21	0.80	[0.55-1.27]	0.4049
p1	Control	50	198	25,25	1.00	-	-
	OIT	44	198	22,22	0.85	[0.53-1.34]	0.554
p2	Control	50	198	25,25	1.00	-	-
	OIT	46	198	23,23	0.90	[0.57-1.42]	0.725
p3	Control	48	198	24,24	1.00	-	-
	OIT	44	198	22,22	0.89	[0.55-1.42]	0.721

OIT: Oil injection technique, p0: nulliparous, p1: uniparous, p2: biparous, p3: triparous, Nb: number, IC: Confidence interval, p: p-value, OR: odds ratio.

### Influence of ovaries development on the determination of the number of egg-laying of anopheles using conventional ovaries dilaceration technique and oil injection into ovaries

Out of 200 mosquitoes examined with Polovodova conventional ovaries dilacerations method, a failure rate of 56% (n = 112) of egg-laying was observed (Table 3). This rate was similar to all ovaries development stages (p = 0.4948). However, a significant difference was observed (p = 0.0274) with oil injection technique (Table 3). We noted that these failures did not depend on the development level of the ovaries.

**Table 3:** **Relation between Christophers’ ovarian development stages and the the number of egg-laying undetermined by Polovodova method based on oil injection and conventional dilaceration techniques**

	**CDT**				**OIT**			
**Stage**	**N Ind**	**Total**	**% Ind**	**P**	**N Ind**	**Total**	**% Ind**	**P**
I	11	25	44.00	0.4948	6	19	31.58a	0.0274
II	88	149	59.06		13	152	08.55b	
III	9	18	50.00		1	20	05.00b	
IV	4	8	50.00		2	7	28.57a	
Total	112	200	56		22	198	11.11	

CDT: Conventional ovries dilaceration technique; OIT: Oil injection technique; Ind: indetermination; N: Number; p = p-value; I, II, III et IV: Christophers’ ovarian development stages.

### Variation of *Anopheles gambiae s.s.* infectivity to *Plasmodium falciparum* according to physiological age

In Adjarra and Ifangni, the infected *Anopheles gambiae s.s.* were those that have laid eggs at least 2 times (Table 4). The infectivity rate of biparous *An. gambiae* mosquitoes was 35.75% in Adjarra. This rate was high in triparous mosquitoes (66.67%) (Table 4). A high infectivity rate was observed in old *Anopheles gambiae s.s*. The same observation was made in Ifangni where the infectivity rate was respectively 33.33% for biparous and 100% for triparous mosquitoes (Table 4).

**Table 4:** **Relation between physiological age and infectivity to** ***P. falciparum*** **of human landing** ***An. gambiae s.s.*** **females caught at Adjarra and at Ifangni**

**egg-laying number**	**Adjarra**				**Ifangni**			
	**n**	**CS+**	**%CS+**	**IC**	**n**	**CS+**	**%CS+**	**IC**
0	18	0	0	[0–18,55]	24	0	0	[0–14,25]
1	81	0	0	[0–4,45]	86	0	0	[0–4,20]
2	14	5	35,75	[12,75-64,86]	21	7	33,33	[14,58-56,97]
3	3	2	66,67	[9,43-98,16]	4	4	100	[39,75-100]
Total	116	7	06,03	[2,46-12,04]	135	11	08,14	[04,14-14,11]

n = Mosquitoes number, CS + = Positivity to circumsporozoïtique antigen, IC = Confidence interval, %CS + = Infectivity rate, 0: nulliparous, 1: uniparous, 2: biparous, 3: triparous.

A significant increase of infectivity with parity level was observed in Ifangni (OR = 5.65 [4.68, 6.81], p < 0.0001) and-Adjarra (OR = 6.02 [4.62, 7.85], P < 0.0001).

## Discussion

The application of oil injection and conventional ovaries dilaceration techniques showed the importance of follicular dilatations in the determination of physiological age in *An. gambiae s.s*. Despite difficulties related to oil injection into ovaries, this technique was accurate for the determination of the number of egg-laying in mosquitoes. A positive correlation was obtained between the number of egg-laying and the number of follicular dilatations observed after oil injection technique in ovaries [16,34]. The efficacy of oil injection technique is related to obtaining intact ovarioles made possible by the use of paraffin oil in inter-ovarian spaces. In addition to isolation failure observed in nulliparous females at the beginning of stage I-II, we noted some under estimation of the number of egg-laying in parous females. This observation was also reported by Rosay [35], and Hoc and Wilkes [16]. Several studies showed the fragility of ovarioles pedicel, susceptible to being broken by needles during isolation of ovaries [22,36,37]. In this condition, it is normal that, confirmed multiparous females appear as uniparous or nulliparous [20]. The origin of the overestimation of the number of egg-laying observed needs to be clarified [35,38,39]. However, the follicular degenerative origin of dilations often occurred at stage II-III during the gonotrophic cycle [40,41]. According to some authors [16], the degenerative cycles observed during subsequent gonotrophic cycles justify the overestimation of recorded numbers of egg-laying. Overall, over and under estimations of the number of egg-laying were very few (1 to 3 for 50 mosquitoes) in our study samples [34]. As previously reported from other studies, ovarioles on which the number of dilatations overlaps exactly to the number of egg-laying are few in mosquitoes [42-44]. Therefore, if Polovodova method is applicable by oil injection for the estimation of egg-laying in *An. gambiae s.s*, it is necessary to make a meticulous reading of the majority of ovarioles before counting the number of egg-laying mosquitoes.

The extreme fragility of pedicle was the cause of inefficacy of conventional ovaries dilaceration, mainly in multiparous mosquitoes. In these mosquitoes, the pedicles lengthen with the increase of dilatations number. This fact does not often allow the isolation of intact ovarioles during classical dilaceration.

Application of oil injection technique was not easy and appeared impossible at the beginning of this study. It was difficult to introduce the tip of the micropipette into the common external lumen of the oviduct before introducing paraffin oil. This technique is usually applied by experts. This explains the small rate of mosquitoes examined by oil injection technique (6 to 8 specimens per hour) during dissection session. According to Beklemishev *et al.,* [45], for systematic use in evaluation of the efficacy of malaria control tools, a physiological age estimation method must be easy and fast. The receptivity of injected oil is relatively low in nulliparous females and did not facilitate the isolation of ovarioles at the beginning of the development of mosquitoes. More likely, the passage of eggs laid could allow opening of calyx light and of common oviduct in parous females. In case of conventional ovaries dilaceration, it requests some manual dexterity in mosquitoes which are in Christopher’s stage I-II mean. At the beginning of their development, the ovarioles are mostly stacked together until their isolation requests more applications. This phenomenon can justify failures of some ovarioles isolation observed in stage I and II during the study.

For mosquitoes collected using human landing catch, only those that have laid at least two times were found positive to *P. falciparum* in both districts of the study. This is justified by the extrinsic incubation time duration of *P. falciparum* which is 10 to 12 days in *An. gambiae s.s.* before mosquitoes become infectious [46]. This duration is due to the gonotrophic cycle time which is 4 to 5 days for the first egg-laying in *An. gambiae s.l.* [47]. Hamon [36] reported that Anopheles mosquitoes cannot transmit malaria when they have less than two weeks, because the percentage of human carriers of gametocytes is low and it is also rare for the first blood meals to be infectious. This idea explains the reason why infectivity rate was high in biparous and triparous mosquitoes in Adjarra and Ifangni. These results confirm the hypothesis that the likelihood for *An. gambiae* mosquitoes of being infected increases with the number of blood meals and the number of egg-laying [45].

## Conclusion

Polovodova classical ovaries dilaceration method is not indicated for the determination of egg-laying in *An. gambiae s.s*. Polovodova oil injection method is more effective but more difficult to handle. Overall, the number of dilatations observed on ovarioles was confirmed with the number of egg-laying of mosquitoes. The use of oil injection technique allowed us to demonstrate that vectors’ likelihood of being infected increases with the number of gonotrophic cycles. Infectious mosquitoes observed are those that have laid eggs at least 2 times.

## Additional file

Additional file 1:
**Tableau Structure of physiological age of wild anopheles reared at insectarium.**
