# Supplementary material for: Application of Polovodova’s method for the determination of physiological age and relationship between the level of parity and infectivity of Plasmodium falciparum in Anopheles gambiae s.s, south-eastern Benin
Source: Parasit Vectors. 2015 Feb 22;8:117. doi: 10.1186/s13071-015-0731-7 (PMC4340486; doi:10.1186/s13071-015-0731-7)
Supplement: Additional file 1: — Tableau Structure of physiological age of wild anopheles reared at insectarium. [file 13071_2015_731_MOESM1_ESM.docx]

**Tableau**: Structure of physiological age of wild anopheles reared at insectarium

|  |  |  | **Method of age (Lot 1)** |  |  | **Method of age (Lot 2)** |  |  |  |  | **Method of age (Lot 1)** |  |  | **Method of age (Lot 2)** |
| --- | --- | --- | --- | --- | --- | --- | --- | --- | --- | --- | --- | --- | --- | --- |
| **Age** | **N°** | **Stages** | **Polovodova (DO)** |  | **Stages** | **Polovodova (TI)** |  | **Age** | **N°** | **Stages** | **Polovodova (DO)** |  | **Stages** | **Polovodova (TI)** |
| **n** | 1 | I | n |  | IId | n |  | **p1** | 1 | I | p1 |  | IId | p1 |
|  | 2 | IId | n |  | I | n |  |  | 2 | IId | n |  | IId | p1 |
|  | 3 | IIf | ? |  | IIf | n |  |  | 3 | IId | p1 |  | IIm | p1 |
|  | 4 | III | n |  | IIm | n |  |  | 4 | IIm | p1 |  | I | ? |
|  | 5 | I | ? |  | III | n |  |  | 5 | IIf | p1 |  | III | p1 |
|  | 6 | IId | n |  | I | ? |  |  | 6 | IIm | p1 |  | IId | p1 |
|  | 7 | IIf | n |  | IId | n |  |  | 7 | IId | p1 |  | IV | p1 |
|  | 8 | III | n |  | IId | n |  |  | 8 | IIf | p2 |  | IIf | p1 |
|  | 9 | IId | n |  | I | n |  |  | 9 | IId | n |  | IIm | n |
|  | 10 | I | ? |  | IId | ? |  |  | 10 | IIm | p1 |  | IId | p1 |
|  | 11 | IIf | n |  | I | n |  |  | 11 | IIm | n |  | IId | p1 |
|  | 12 | IId | n |  | IIm | n |  |  | 12 | IIf | p1 |  | IIf | p1 |
|  | 13 | III | n |  | IIf | n |  |  | 13 | IId | p1 |  | IIm | p1 |
|  | 14 | IId | n |  | I | n |  |  | 14 | I | p1 |  | IIf | p1 |
|  | 15 | I | ? |  | III | n |  |  | 15 | IId | p1 |  | III | p1 |
|  | 16 | I | n |  | I | n |  |  | 16 | IId | p1 |  | IId | p1 |
|  | 17 | IV | n |  | IId | ? |  |  | 17 | IIf | p1 |  | IIm | p1 |
|  | 18 | I | ? |  | IId | n |  |  | 18 | III | p1 |  | I | p1 |
|  | 19 | IId | n |  | IIm | n |  |  | 19 | I | p1 |  | IId | p1 |
|  | 20 | I | n |  | IId | n |  |  | 20 | IId | p1 |  | IId | p1 |
|  | 21 | IId | n |  | I | n |  |  | 21 | IId | p1 |  | IIm | p1 |
|  | 22 | I | n |  | IIm | n |  |  | 22 | IIm | p1 |  | IIf | p2 |
|  | 23 | IIm | ? |  | IId | n |  |  | 23 | IId | n |  | IId | p1 |
|  | 24 | IId | n |  | I | ? |  |  | 24 | IIf | p1 |  | IId | p1 |
|  | 25 | I | ? |  | IV | n |  |  | 25 | IId | p1 |  | IIm | p1 |
|  | 26 | IId | n |  | III | n |  |  | 26 | I | p1 |  | III | p2 |
|  | 27 | I | n |  | I | ? |  |  | 27 | IId | p1 |  | IId | p1 |
|  | 28 | IIm | n |  | IIf | n |  |  | 28 | III | p1 |  | IId | p1 |
|  | 29 | III | n |  | IId | n |  |  | 29 | IId | n |  | IIm | p1 |
|  | 30 | IIf | n |  | I | n |  |  | 30 | IV | p1 |  | IIm | n |
|  | 31 | I | ? |  | III | n |  |  | 31 | IIm | p1 |  | IId | p1 |
|  | 32 | IIf | n |  | IId | ? |  |  | 32 | IId | p1 |  | IId | p1 |
|  | 33 | I | n |  | IId | p1 |  |  | 33 | IIf | p1 |  | IIm | p1 |
|  | 34 | IId | n |  | IIm | n |  |  | 34 | IId | p1 |  | IId | p1 |
|  | 35 | IId | n |  | IIf | n |  |  | 35 | IId | n |  | IId | p1 |
|  | 36 | IIm | ? |  | I | n |  |  | 36 | IIm | p1 |  | IId | p1 |
|  | 37 | I | ? |  | IId | n |  |  | 37 | IId | p1 |  | IV | p1 |
|  | 38 | IIm | n |  | I | n |  |  | 38 | IIm | p1 |  | IId | p1 |
|  | 39 | IIm | n |  | IIm | n |  |  | 39 | IIf | p1 |  | IId | p1 |
|  | 40 | I | n |  | III | n |  |  | 40 | IId | p1 |  | IIf | p1 |
|  | 41 | IIf | n |  | IIf | n |  |  | 41 | IIm | p1 |  | IId | p1 |
|  | 42 | IId | n |  | IV | p1 |  |  | 42 | IId | p1 |  | IIm | p2 |
|  | 43 | III | n |  | IId | n |  |  | 43 | III | p1 |  | IId | p1 |
|  | 44 | I | ? |  | IId | n |  |  | 44 | IId | p1 |  | IIf | p1 |
|  | 45 | IV | n |  | I | ? |  |  | 45 | IIm | p1 |  | IId | p1 |
|  | 46 | IId | n |  | IIf | n |  |  | 46 | IId | p1 |  | IId | p1 |
|  | 47 | IId | n |  | IId | n |  |  | 47 | IId | p1 |  | III | p1 |
|  | 48 | I | ? |  | III | n |  |  | 48 | IIf | p1 |  | IId | p1 |
|  | 49 | IId | n |  | I | n |  |  | 49 | I | p1 |  | IIm | p1 |
|  | 50 | I | n |  | IId | n |  |  | 50 | IIm | n |  | IId | p1 |
| **p2** | 1 | IId | p1 |  | IIm | p2 |  | **p3** | 1 | IIm | p1 |  | IId | p3 |
|  | 2 | IId | p1 |  | IIm | p2 |  |  | 2 | IId | p1 |  | IIm | p3 |
|  | 3 | IIm | n |  | IId | p2 |  |  | 3 | IId | p1 |  | IIm | p3 |
|  | 4 | IId | p1 |  | III | p2 |  |  | 4 | IIm | p1 |  | III | p3 |
|  | 5 | IIf | p1 |  | IIf | p2 |  |  | 5 | IV | p1 |  | IIf | p4 |
|  | 6 | IId | p1 |  | IV | p3 |  |  | 6 | III | p1 |  | IIm | p3 |
|  | 7 | IId | p1 |  | IIm | p2 |  |  | 7 | IId | p2 |  | IIm | p3 |
|  | 8 | IIm | p1 |  | IId | p2 |  |  | 8 | IIf | p1 |  | III | p3 |
|  | 9 | III | p2 |  | IIf | p2 |  |  | 9 | IId | p1 |  | IIm | p3 |
|  | 10 | IV | p2 |  | IIm | p2 |  |  | 10 | IId | n |  | IId | p3 |
|  | 11 | IId | p1 |  | I | p2 |  |  | 11 | IV | p2 |  | IIm | p3 |
|  | 12 | III | p1 |  | IIm | p2 |  |  | 12 | IId | p1 |  | IId | p2 |
|  | 13 | IId | p1 |  | IIf | p2 |  |  | 13 | III | p1 |  | IIf | p3 |
|  | 14 | IId | p1 |  | IIm | p2 |  |  | 14 | IIm | p1 |  | IIm | p1 |
|  | 15 | IIm | p1 |  | IId | p2 |  |  | 15 | IId | p1 |  | IIm | p3 |
|  | 16 | IId | p1 |  | IIm | p2 |  |  | 16 | IIf | p2 |  | IId | p3 |
|  | 17 | IV | p1 |  | IIm | p2 |  |  | 17 | III | p2 |  | IIf | p3 |
|  | 18 | IIf | p1 |  | IId | p2 |  |  | 18 | IId | p1 |  | IIm | p3 |
|  | 19 | IIm | p1 |  | IIf | p2 |  |  | 19 | IIm | p1 |  | IIf | p3 |
|  | 20 | IId | p1 |  | IIf | p2 |  |  | 20 | IId | p1 |  | IIf | p3 |
|  | 21 | IId | p2 |  | IIm | p2 |  |  | 21 | IIm | p2 |  | IIm | p3 |
|  | 22 | IId | p1 |  | IId | p2 |  |  | 22 | IIf | p3 |  | IId | p3 |
|  | 23 | IIm | p1 |  | IIm | p2 |  |  | 23 | IId | p1 |  | IIm | p3 |
|  | 24 | IId | p1 |  | III | p2 |  |  | 24 | IId | p1 |  | IId | p3 |
|  | 25 | I | p1 |  | IId | p2 |  |  | 25 | IIm | p1 |  | IIf | p3 |
|  | 26 | IId | n |  | IIm | p2 |  |  | 26 | IId | p1 |  | IIm | p3 |
|  | 27 | IId | p1 |  | IId | p2 |  |  | 27 | IIm | p1 |  | IId | p3 |
|  | 28 | IIm | p1 |  | IIm | p2 |  |  | 28 | IIf | p1 |  | IIm | p3 |
|  | 29 | IId | p1 |  | IId | p2 |  |  | 29 | IId | p2 |  | IIf | n |
|  | 30 | IId | p1 |  | IIf | p2 |  |  | 30 | IIf | p1 |  | IId | p3 |
|  | 31 | III | p1 |  | IIm | p2 |  |  | 31 | IIf | p1 |  | III | p3 |
|  | 32 | IId | p2 |  | IIm | p2 |  |  | 32 | IV | p1 |  | IId | p3 |
|  | 33 | III | p1 |  | III | p2 |  |  | 33 | IIf | p1 |  | IIm | p3 |
|  | 34 | IId | p1 |  | IIm | p2 |  |  | 34 | IId | p1 |  | IIm | p3 |
|  | 35 | IIf | p1 |  | IIm | p2 |  |  | 35 | IId | p1 |  | IId | p3 |
|  | 36 | IIm | p1 |  | IId | p1 |  |  | 36 | IIf | p1 |  | IId | p3 |
|  | 37 | IId | p1 |  | IV | p2 |  |  | 37 | IIf | p2 |  | IIf | p3 |
|  | 38 | IIf | p2 |  | IId | p2 |  |  | 38 | IIm | p1 |  | III | p3 |
|  | 39 | IId | p1 |  | IIm | p2 |  |  | 39 | III | p1 |  | IIm | p3 |
|  | 40 | I | p2 |  | IIf | p3 |  |  | 40 | IId | n |  | IIf | p3 |
|  | 41 | III | p1 |  | IId | p2 |  |  | 41 | IIf | p1 |  | IId | p3 |
|  | 42 | IId | p1 |  | IIm | p2 |  |  | 42 | IIm | p1 |  | IIm | p3 |
|  | 43 | IIm | p1 |  | IId | p2 |  |  | 43 | IId | p1 |  | III | p3 |
|  | 44 | IId | p1 |  | III | p2 |  |  | 44 | IIf | p1 |  | IId | p3 |
|  | 45 | IId | n |  | IId | p2 |  |  | 45 | IId | p1 |  | I | p3 |
|  | 46 | IIf | p1 |  | IIf | p2 |  |  | 46 | IIm | p2 |  | IId | p3 |
|  | 47 | IIm | p2 |  | I | n |  |  | 47 | IIm | p1 |  | IIf | p3 |
|  | 48 | IIm | p1 |  | III | p2 |  |  | 48 | III | p1 |  | IV | p3 |
|  | 49 | I | p1 |  | IIm | p2 |  |  | 49 | IId | p1 |  |  |  |
|  | 50 | IIf | p1 |  | IIm | p2 |  |  | 50 | IId | p1 |  |  |  |

**DO** : Ovaries conventional dilaceration ; **TI** : Oil injection ; **n** : nulliparous ; **p1** : uniparous ; **p2** : biparous; **p3**: triparous ; **p4** : quadriparous ; **I**, **IId** (begenning), **IIm** (mean), **IIf** (end), **III** and **IV** : development stages of ovaries **?**: Conventional ovaries dilaceration fail / fail of oil injection in ovaries / illegibility
